# Emergence of OXA-48-producing *Klebsiella pneumoniae* in Lithuania, 2023: a multi-cluster, multi-hospital outbreak

**DOI:** 10.2807/1560-7917.ES.2024.29.16.2400188

**Published:** 2024-04-18

**Authors:** Paulius Greičius, Marius Linkevicius, Jelena Razmuk, Jekaterina Sinotova, Erik Alm, Olov Svartström, Valeria Bortolaia, Eglė Kudirkienė, Louise Roer, Rene S Hendriksen, Gabija Tamoliūnaitė, Daniel Palm, Dominique L Monnet, Anke Kohlenberg, Algirdas Griškevičius

**Affiliations:** 1 National Public Health Surveillance Laboratory (NVSPL), Vilnius, Lithuania; 2 European Centre for Disease Prevention and Control (ECDC), Stockholm, Sweden; 3 Statens Serum Institut (SSI), Copenhagen, Denmark; 4 Technical University of Denmark, National Food Institute (DTU Food), Kongens Lyngby, Denmark

**Keywords:** carbapenem-resistant Enterobacterales, *Klebsiella pneumoniae*, carbapenemase, OXA-48, surveillance, whole genome sequencing

## Abstract

In 2023, an increase of OXA-48-producing *Klebsiella pneumoniae* was noticed by the Lithuanian National Public Health Surveillance Laboratory. Whole genome sequencing (WGS) of 106 OXA-48-producing *K. pneumoniae* isolates revealed three distinct clusters of carbapenemase-producing *K. pneumoniae* high-risk clones, including sequence type (ST) 45 (n = 35 isolates), ST392 (n = 32) and ST395 (n = 28), involving six, six and nine hospitals in different regions, respectively. These results enabled targeted investigation and control, and underscore the value of national WGS-based surveillance for antimicrobial resistance.

Implementation of whole genome sequencing (WGS) for surveillance and control of multidrug-resistant bacteria was initiated at the Lithuanian National Public Health Surveillance Laboratory (NVSPL) in 2023. Among carbapenem-resistant *Klebsiella pneumoniae* isolates routinely referred from clinical laboratories, NVSPL noticed a sudden increase of isolates carrying *bla*_OXA-48-like_ in 2023. To validate laboratory procedures while generating real-time genomic data for public health purposes, 106 isolates of *K. pneumoniae *carrying *bla*_OXA-48-like_ were selected for a pilot study. The aim of this study was to determine the genetic relatedness of isolates for tracking transmission pathways in involved hospitals for improved infection prevention and control (IPC) measures and to describe the molecular characteristics of the involved clones. Here, we report preliminary epidemiological, microbiological and genomic findings from the ongoing OXA-48-producing *K. pneumoniae* outbreak.

## Data collection and analysis

Submission of carbapenem-resistant Enterobacterales (CRE) for reference testing to NVSPL is mandatory in Lithuania since 2014 [1]. Confirmatory antimicrobial susceptibility testing and carbapenemase gene detection by PCR of *bla*_OXA-48-like_, *bla*_KPC_, *bla*_NDM_ and *bla*_VIM_ genes is routinely performed at NVSPL. For this investigation, minimal inhibitory concentrations (MICs) for meropenem were determined using Bruker Micronaut-S plates and interpreted according to the European Committee on Antimicrobial Susceptibility Testing (EUCAST) clinical breakpoints [2].

Of 308 *K. pneumoniae* isolates confirmed by NVSPL to carry *bla*_OXA-48-like_ genes in 2022–23, 106 (34.4%) were selected for sequencing. These isolates originated from clinical samples collected from 17 public hospitals between 28 April 2022 and 29 November 2023, covering the period of emergence. At least one isolate was selected per hospital. Supplementary Figure S1 provides time distribution of these isolates by sequencing status and major clusters. It also contains information on the isolate selection. 

Paired-end sequencing was performed using Illumina platform. Reads were processed in Ridom SeqSphere v9.0.10 [3] using trimming parameters of ≥ 30 average quality in a window of 20 bases and SKESA assembler [4] and subjected to quality control. The Institute Pasteur scheme was used for typing [5]. Resistance genes were identified using NCBI AMRFinderPlus [6] within SeqSphere and Kleborate v2.4.1 [7] with standard parameters. A minimum spanning tree based on core genome multilocus sequence typing (cgMLST) scheme [3] was constructed in SeqSphere with samples that contained < 95% cgMLST target loci excluded. Clusters were determined with a cut-off of ≤ 5 allelic differences (ADs).

## Detection of carbapenemase genes over time

The carbapenemase gene distribution of submitted Enterobacterales isolates determined by NVSPL during 2017–23 is shown in the Table. Carbapenemase-producing Enterobacterales were rare in Lithuania until 2019, when a large outbreak of *K. pneumoniae* carrying *bla*_KPC-2_ occurred (described in [8]). After the implementation of control measures, the number of identified carbapenemase-producing *K. pneumoniae* (CPKP) isolates declined but remained at a higher level than before the outbreak. In 2023, an increase in Enterobacterales carrying *bla*_OXA-48-like_ (n = 319), mainly *K. pneumoniae* (n = 302, 94.7%), was identified (Table).

**Table:** Isolates of Enterobacterales carrying carbapenemase gene(s) confirmed by PCR, Lithuania, 2017–2023 (n = 882)

Carbapenemase gene	2017	2018	2019	2020	2021	2022	2023	Total
*bla*_KPC_	2	1	359	58	13	16	17	466
*bla*_OXA-48-like_	0	2	0	0	0	3	294	299
*bla*_NDM_	8	2	0	6	9	5	49	79
*bla*_OXA-48-like_ + *bla*_NDM_	0	0	0	0	0	3	25	28
*bla*_VIM_	2	0	0	1	2	0	2	7
*bla*_VIM_ + *bla*_NDM_	0	0	0	0	0	3	0	3
Total	12	5	359	65	24	30	387	882

*bla*: beta-lactamase; KPC: *Klebsiella pneumoniae* carbapenemase; NDM: New Delhi metallo-beta-lactamase; OXA-48-like: Oxacillinase-48-like; VIM: Verona integron-encoded metallo-beta-lactamase.

## Distribution of sequence types of *Klebsiella pneumoniae* carrying *bla*_OXA-48_

**Figure 1:**
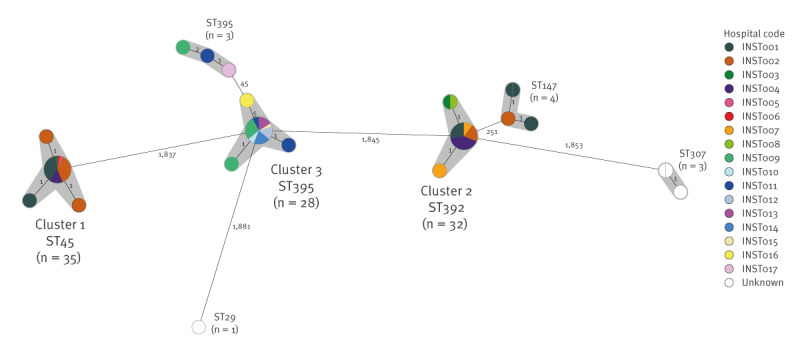
Clusters of OXA-48-producing *Klebsiella pneumoniae* isolates by hospital, Lithuania, 2022–2023 (n = 106 isolates)

AD: allelic difference; ST: sequence type.

The 106 sequenced *K. pneumoniae* isolates carrying *bla*_OXA-48_ belonged to six *K. pneumoniae* sequence types (ST). The most frequent ST was ST45 (n = 35), followed by ST392 (n = 32) and ST395 (n = 31). A few isolates of other STs, i.e. ST147 (n = 4), ST307 (n = 3), ST29 (n = 1), also harboured *bla*_OXA-48_ and four of these (ST307, n = 3; ST147, n = 1) co-carried *bla*_NDM-1_. Six clusters of suspected recent transmission were identified (Figure 1), including three large multi-hospital clusters with > 20 isolates (Cluster 1–3), described in further detail below.

The numbers on the branches represent the number of ADs between isolates; nodes with partition represent more than one isolate with 0 AD. Genetic clusters are shaded in grey.

Reads were processed in Ridom SeqSphere v9.0.10 [3] using trimming parameters of ≥ 30 average quality in a window of 20 bases and SKESA assembler [4]. The quality control parameters were a minimum mash identity score of 0.95, genome coverage ≥ 30 x, assembly contig number < 500, sequence length of the shortest contig for covering 50% of the total assembly length (N50) > 15,000 base pairs, and assembly size within 10% of expected *K. pneumoniae* genome size of 5.5 Mbs [18]. The Institute Pasteur scheme was used for typing [5]. A minimum spanning tree based on core genome multilocus sequence typing (cgMLST) scheme [3] was constructed in SeqSphere with samples that contained < 95% cgMLST target loci excluded. Clusters were determined with a cut-off of ≤ 5 allelic differences (ADs).

## Multi-hospital clusters

**Figure 2:**
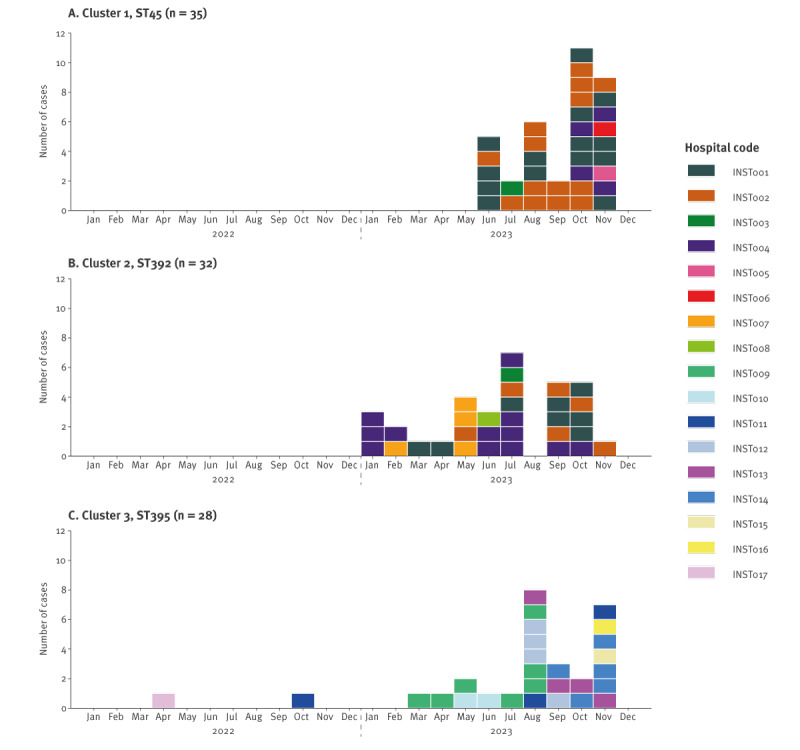
Epidemic curves for the three major *Klebsiella pneumoniae* clusters by hospital, Lithuania, 2022–2023 (n = 95 cases)

**Figure 3:**
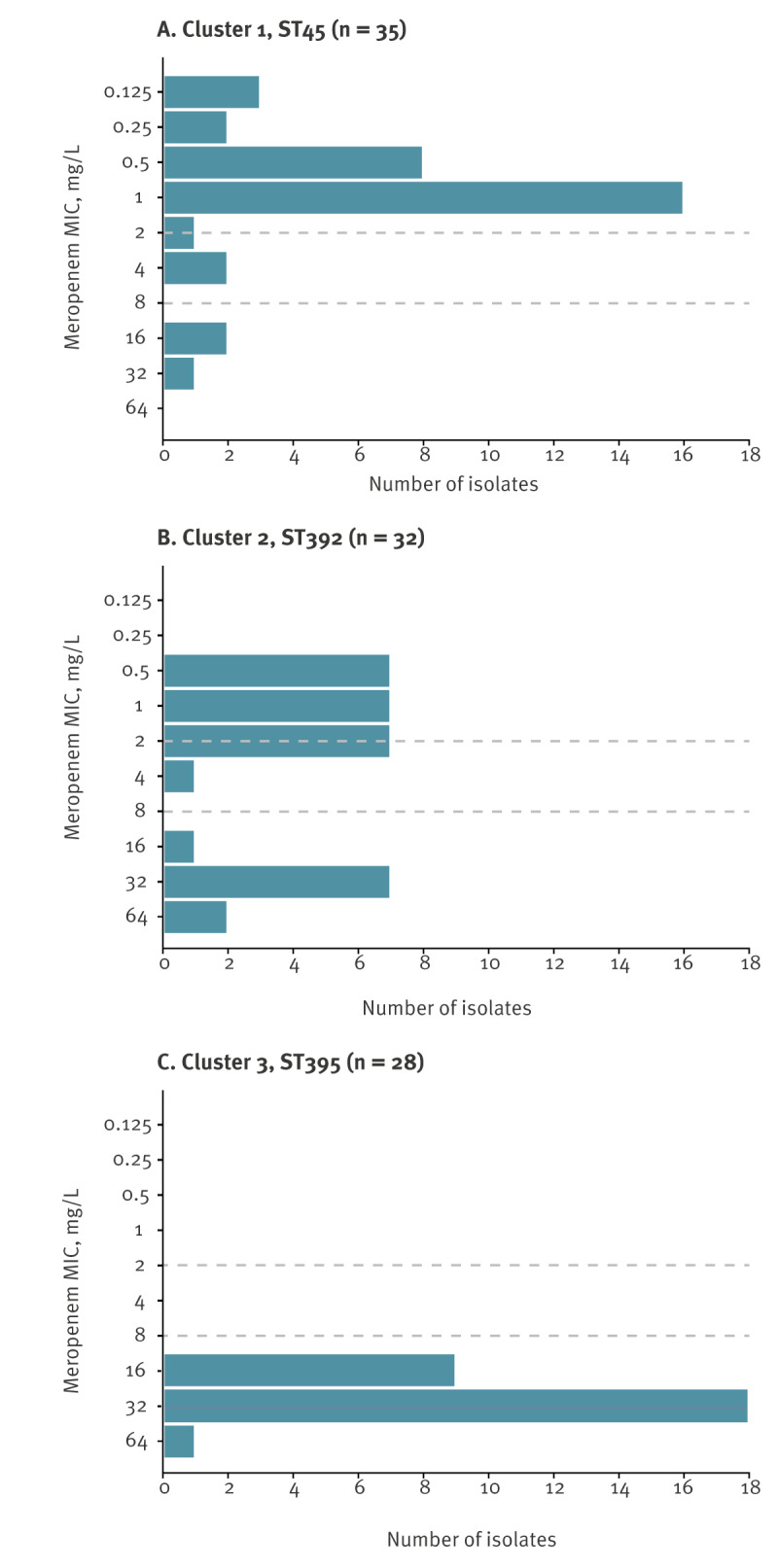
Meropenem minimal inhibitory concentration values for *Klebsiella pneumoniae* isolates from the three major clusters, Lithuania, 2022–2023 (n = 95 isolates)

MIC: minimal inhibitory concentration; ST: sequence type.

Cluster 1 included 35 isolates of *K. pneumoniae* ST45 involving six hospitals from two counties, indicating interregional spread in central-eastern Lithuania including the capital region. The first isolate was detected in June 2023 in hospital INST001 (Figure 2A) and 10 isolates were from blood samples. While no extended-spectrum beta-lactamase genes were detected, 25 isolates carried the plasmid-mediated AmpC gene *bla*_DHA-1_. Three isolates were resistant to meropenem (Figure 3A) of which one had a truncated outer membrane porin (OMP), OmpK36.

ST: sequence type.

Grey lines indicate European Committee on Antimicrobial Susceptibility Testing (EUCAST) clinical breakpoints [2] for meropenem in Enterobacterales: susceptible ≤ 2 mg/L, resistant > 8 mg/L.

Cluster 2 included 32 isolates of *K. pneumoniae* ST392 involving six hospitals from three counties indicating interregional spread in central, northern and eastern Lithuania including the capital region. The first isolate was detected in hospital INST004 in January 2023 (Figure 2B) and 16 isolates were from blood samples. Twenty-nine of these isolates also carried the *bla*_CTX-M-15_ gene. Ten isolates were resistant to meropenem (Figure 3B), of which nine had truncated OMPs, either OmpK36 (n = 8) or OmpK35 (n = 1).

Cluster 3 included 28 isolates of *K. pneumoniae* ST395 involving nine hospitals from four counties indicating interregional spread in western Lithuania. The first isolate was detected in April 2022 in hospital INST017 (Figure 2C) and three isolates were from blood samples. All isolates carried truncated OmpK35 with duplication of OmpK36 Gly134-Asp135 (OmpK36GD) and were resistant to meropenem (Figure 3C). Additionally, 18 of these isolates carried the *bla*_CTX-M-15_ gene and one isolate the *bla*_CTX-M-3_ gene.

For Clusters 1, 2 and 3, the median ages of patients were 75 (range: 36–97), 64 (range: 29–94) and 74 (range: 30–88) years and the male-to-female ratios were 1.3, 0.9 and 1.5, respectively. Sample types varied by cluster, and the distribution is provided in Supplementary Figure S2. The 17 hospitals with confirmed *K. pneumoniae* isolates carrying *bla*_OXA-48_ represent 26.6% of public general hospitals (n = 64) from 7 of 10 counties. Four hospitals (INST001–004) had isolates in both Cluster 1 and Cluster 2 while there was no overlap with any hospital with isolates in Cluster 3 (Figure 2). Information on patient transfer was not available at the time of this report.

## Control measures

In response to detection of the clusters, a national working group for investigation of antimicrobial resistance (AMR) was established. Hospitals were requested to collect retrospective epidemiological information on cases in their institutions and potential links to other healthcare facilities, and to provide information on their capacity for screening and isolation. The working group recommended enhanced IPC measures for cases with CRE, including isolation and investigation of contact patients and is presently drafting a guidance document to reduce the spread of CRE in healthcare facilities in Lithuania. Sequencing of *K. pneumoniae* isolates carrying *bla*_OXA-48-like_ genes continues at NVSPL.

## Discussion

The worldwide spread of CPKP is driven by the transmission of high-risk clones in healthcare facilities [9,10]. A large outbreak of *K. pneumoniae* carrying *bla*_KPC-2_ occurred in Lithuania in 2019 [8]. At that time, the increase of CPKP was largely based on clonal spread of *K. pneumoniae* ST392 and spread of an IncN plasmid harbouring *bla*_KPC-2_ to other *K. pneumoniae* STs and Enterobacterales species [8]. The outbreak mainly occurred in one hospital with limited spread to other healthcare facilities via patient transfer [8]. In contrast, this genomic investigation highlights the changing epidemiology of CPKP in Lithuania, with diversification of involved high-risk clones and rapid interregional spread throughout the healthcare system in less than one year.

Cluster 1 – the largest cluster – was formed by *K. pneumoniae* ST45, which has been described as a major clone among carbapenem-non-susceptible *K. pneumoniae* isolates [7]; an outbreak of *K. pneumoniae* ST45 carrying *bla*_GES-5/-1_ occurred in a hospital in Poland between 2017 and 2019 [11]. Cluster 2 involved *K. pneumoniae* ST392, which had caused the outbreak in Lithuania in 2019 [8] as well as outbreaks and cross-border spread in other European countries [12,13]. Finally, Cluster 3 consisted of *K. pneumoniae* isolates belonging to ST395, another well-known high-risk clone spreading in various countries [14] including in Eastern Europe [15]; ST395 has also been related to prior healthcare exposure in Ukraine [16]. The large number of bloodstream isolates in Clusters 1 and 2 highlights the clinical relevance of the identified CPKP cases, but also suggests considerable under-detection, e.g. in other types of infection or from carriage, in hospitals in Lithuania.

Isolates in different clusters showed varying levels of meropenem resistance with the highest resistance identified in isolates from Cluster 3 (ST395), which had alterations in major OMPs. Consistent with previous observation [17], combined OmpK35 truncation with OmpK36GD in OXA-48-producing isolates resulted in meropenem MICs exceeding the clinical breakpoint, as evident in isolates from Cluster 3 (ST395). This highlights the ability of CPKP to combine low-level carbapenem resistance mechanisms, i.e. altered OMPs (chromosomal feature) and OXA-48 production (mobile genetic element feature), resulting in clinically relevant phenotypes.

This investigation has been enabled by the capacity building and harmonisation of WGS within the EURGen-RefLabCap project (https://www.eurgen-reflabcap.eu), funded by the European Commission. Lithuania is one of 16 European countries receiving specific training and bespoke advice to conduct genomic pilot studies and one of the first countries for which results have become available. This report may therefore provide an example for other countries currently setting up genomic AMR surveillance.

A limitation of this investigation is that it includes preliminary data from an ongoing outbreak investigation. Hence, at this stage, only epidemiological data collected by NVSPL under national surveillance regulations could be included. Additionally, it is too early to investigate the effectiveness of the implemented IPC measures. Furthermore, only a subset of *K. pneumoniae* isolates carrying *bla*_OXA-48_ has been sequenced and clusters may further increase in size and additional clusters may be detected once more genomic data become available.

## Conclusion

This report demonstrates the value of national WGS-based surveillance for AMR. The newly established WGS capacity for AMR at NVSPL helped to disentangle an increase of *K. pneumoniae* carrying *bla*_OXA-48_ into at least three separate clonal outbreaks, facilitating targeted investigations and the strengthening of national control efforts. This study also highlights the need to remain vigilant of repeated introduction and increasing spread of CPKP high-risk clones in healthcare systems and to ensure that hospitals are prepared to detect CPKP cases early and prevent onward transmission, as highlighted in the ECDC and WHO guidance documents.

## Supplementary Data

384000 Supplement
