# Supplementary material for: Emergence of OXA-48-producing Klebsiella pneumoniae in Lithuania, 2023: a multi-cluster, multi-hospital outbreak
Source: Euro Surveill. 2024 Apr 18;29(16):2400188. doi: 10.2807/1560-7917.ES.2024.29.16.2400188 (PMC11027475; doi:10.2807/1560-7917.ES.2024.29.16.2400188)
Supplement: Supplement [file 24-00188_LINKEVICIUS_Supplement.pdf]

Emergence of OXA-48-producing *Klebsiella pneumoniae* in Lithuania, 2023: a multi-cluster, multi-hospital outbreak

Supplemental material

This supplementary material is hosted by Eurosurveillance as supporting information alongside the article "Emergence of OXA-48-producing *Klebsiella pneumoniae* in Lithuania, 2023: a multi-cluster, multi-hospital outbreak" on behalf of the authors who remain responsible for the accuracy and appropriateness of the content. The same standards for ethics, copyright, attributions and permissions as for the article apply. Eurosurveillance is not responsible for the maintenance of any links or email addresses provided therein.

Supplementary figures

Supplementary Figure S1. Time distribution of *Klebsiella pneumoniae* isolates carrying *bla*$_{\text{OXA-48-like}}$ genes, Lithuania, 2022–2023 (n = 308)

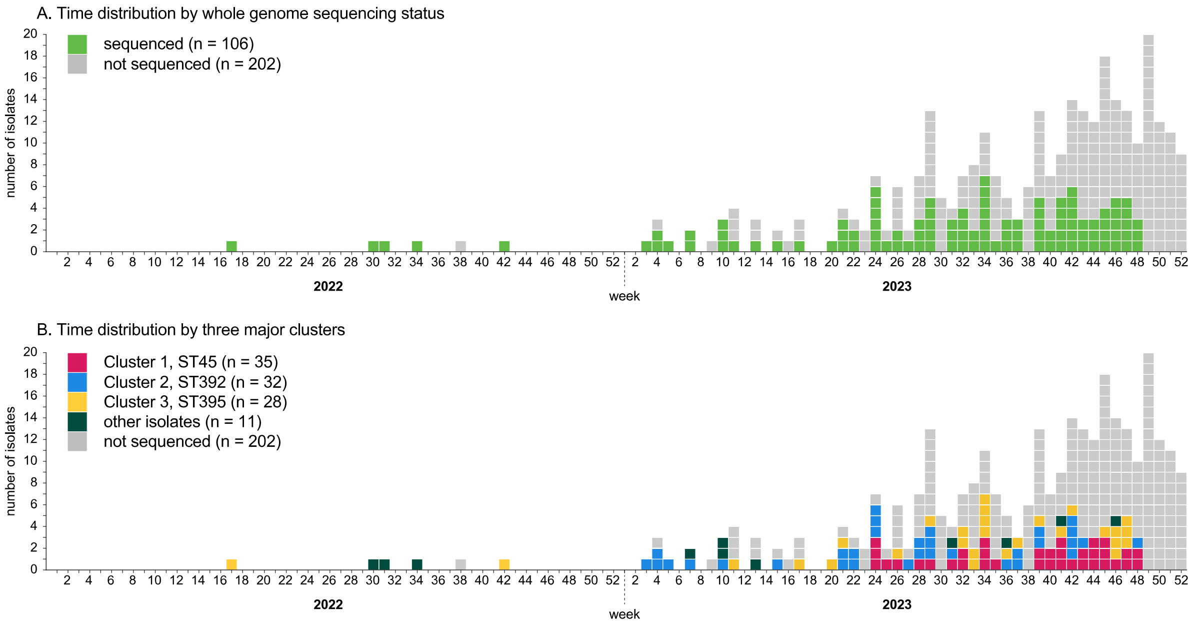

ST: sequence type.

Isolates were selected in a stepwise manner including at least one isolate per involved hospital followed by selection of further isolates for time coverage and proportionate to the total number of isolates per hospital.

Supplementary Figure S2. Sample type distribution of *Klebsiella pneumoniae* isolates carrying *bla*$_{OXA-48}$ in Cluster 1-3, Lithuania, 2022–2023 (n = 95)

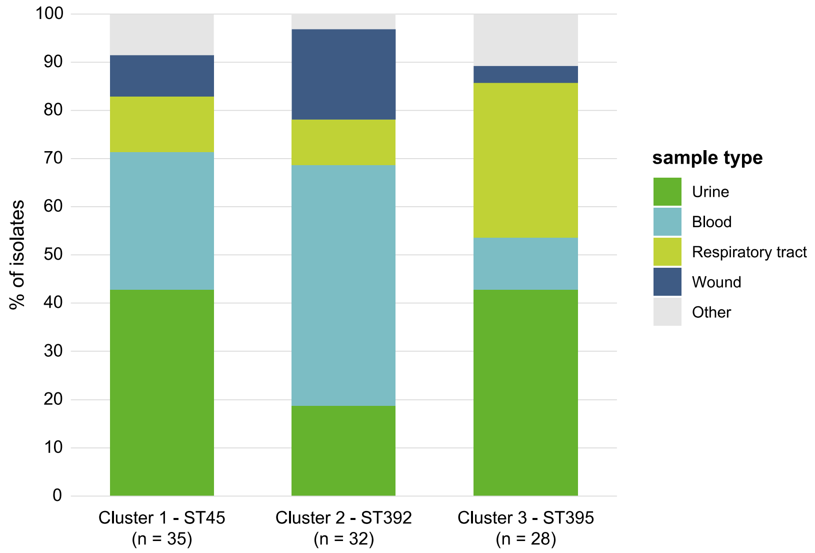

ST: sequence type.
